# Optical Myography: Detecting Finger Movements by Looking at the Forearm

**DOI:** 10.3389/fnbot.2016.00003

**Published:** 2016-04-11

**Authors:** Christian Nissler, Nikoleta Mouriki, Claudio Castellini

**Affiliations:** ^1^Institute of Robotics and Mechatronics, German Aerospace Center (DLR), Wessling, Germany

**Keywords:** rehabilitation robotics, human–machine interface, hand prostheses, computer vision, myography

## Abstract

One of the crucial problems found in the scientific community of assistive/rehabilitation robotics nowadays is that of automatically detecting what a disabled subject (for instance, a hand amputee) wants to do, exactly when she wants to do it, and strictly for the time she wants to do it. This problem, commonly called “intent detection,” has traditionally been tackled using surface electromyography, a technique which suffers from a number of drawbacks, including the changes in the signal induced by sweat and muscle fatigue. With the advent of realistic, physically plausible augmented- and virtual-reality environments for rehabilitation, this approach does not suffice anymore. In this paper, we explore a novel method to solve the problem, which we call Optical Myography (OMG). The idea is to visually inspect the human forearm (or stump) to reconstruct what fingers are moving and to what extent. In a psychophysical experiment involving ten intact subjects, we used visual fiducial markers (*AprilTags*) and a standard web camera to visualize the deformations of the surface of the forearm, which then were mapped to the intended finger motions. As ground truth, a visual stimulus was used, avoiding the need for finger sensors (force/position sensors, datagloves, etc.). Two machine-learning approaches, a linear and a non-linear one, were comparatively tested in settings of increasing realism. The results indicate an average error in the range of 0.05–0.22 (root mean square error normalized over the signal range), in line with similar results obtained with more mature techniques such as electromyography. If further successfully tested in the large, this approach could lead to vision-based intent detection of amputees, with the main application of letting such disabled persons dexterously and reliably interact in an augmented-/virtual-reality setup.

## 1 Introduction

Optical motion tracking and image processing are witnessing an astonishing progress. Cameras offer higher and higher resolutions at cheaper and cheaper prices; new kinds of optical sensors appear, including structured light and (near-)infrared depth sensors; and computer vision, i.e., advanced image processing, offers unheard-of possibilities. We have, nowadays, the concrete chance of building affordable, integrated systems providing complex virtual worlds, in which a human subject can interact essentially without wearing any constraining sensors. Such systems could, in principle, be bundled in a cheap application to be used at home, either as a gaming environment or as a rehabilitation device for the disabled.

In fact, in the field of assistive/rehabilitation robotics, this opens up an interesting possibility: that of using optical tracking and recognition to reconstruct the intended movements of an amputee, just by looking at her stump (intent detection); the idea is that of detecting the deformations induced by muscle activity in the stump and associate them with the movements the subject tries to enforce. This idea is not new; indeed, it has so far been enforced using pressure (Phillips and Craelius, 2005; Yungher et al., 2011; Castellini and Ravindra, 2014) and tactile sensors (Radmand et al., 2014); the advantages of this approach with respect to the more traditional methods of intent detection, such as surface electromyography (sEMG (Zecca et al., 2002; Merletti et al., 2011)), are that this kind of sensors is usually much cheaper than sEMG electrodes and that they enforce a better resilience against the typical pitfalls of sEMG, such as muscle fatigue (Yungher et al., 2011; Ravindra and Castellini, 2014). Another approach is the usage of features extracted from ultrasound images (US) of the forearm (Zheng et al., 2006; Castellini et al., 2012; Castellini and Sierra González, 2013; Sierra González and Castellini, 2013). This approach has the benefit that structural changes of skeletal muscles at different depths can be detected. Furthermore, in Ho et al. (2011), a combination of several features, such as depth maps and silhouettes, is fused to obtain a robust hand and finger tracking algorithm, solving the problem of self-occlusions of the fingers. Nevertheless, to the best of our knowledge, there is as yet no method to obtain information about the motion of the fingers by only using visual information extracted from the forearm. As a matter of fact, the human skin provides very little texture and is therefore very challenging for state-of-the-art image feature detectors/descriptors, such as SIFT (Lowe, 1999) or SURF (Bay et al., 2006), to mention two popular ones, which fail when confronted with reliably identifying and tracking landmarks on the human skin. On the other hand, artificial fiducial markers, such as AprilTags (Olson, 2011), are widely used in, e.g., Augmented Reality (Dong et al., 2013), mobile robotics (Feng and Kamat, 2012), or even camera calibration (Richardson et al., 2013), and proved to be robust and reliable features to track.

Even though attaching markers to the skin is unobtrusive, a system detecting the muscle motion directly by observing the arm would be attractive. In Liu et al. (2005) and Wu et al. (2012), a method to artificially amplify subtle motions in video streams is shown. These methods used to be computation intensive and therefore only available as offline methods, but recently, a real-time application is available (Wadhwa et al., 2014). This method amplifies small motions by a definite factor, but the abovementioned problem of identifying and tracking precise features on the arm still exists. Using plain optical recognition to track the forearm and detect finger movements could, e.g., enforce interaction by an amputee in a virtual world, without using any device on the subject’s body. The usage of human–machine interfaces in virtual-/augmented-reality scenarios is known to alleviate phantom-limb pain and change/improve the phantom feeling (Murray et al., 2007; Ortiz-Catalan et al., 2014; Trojan et al., 2014); mismatch between motor commands and sensory information in motor-disabled patients is conjectured to be at the heart of detrimental phantom feelings and phantom-limb pain (Flor et al., 1995; Flor and Birbaumer, 2000; Diers et al., 2010).

**Figure 1:**
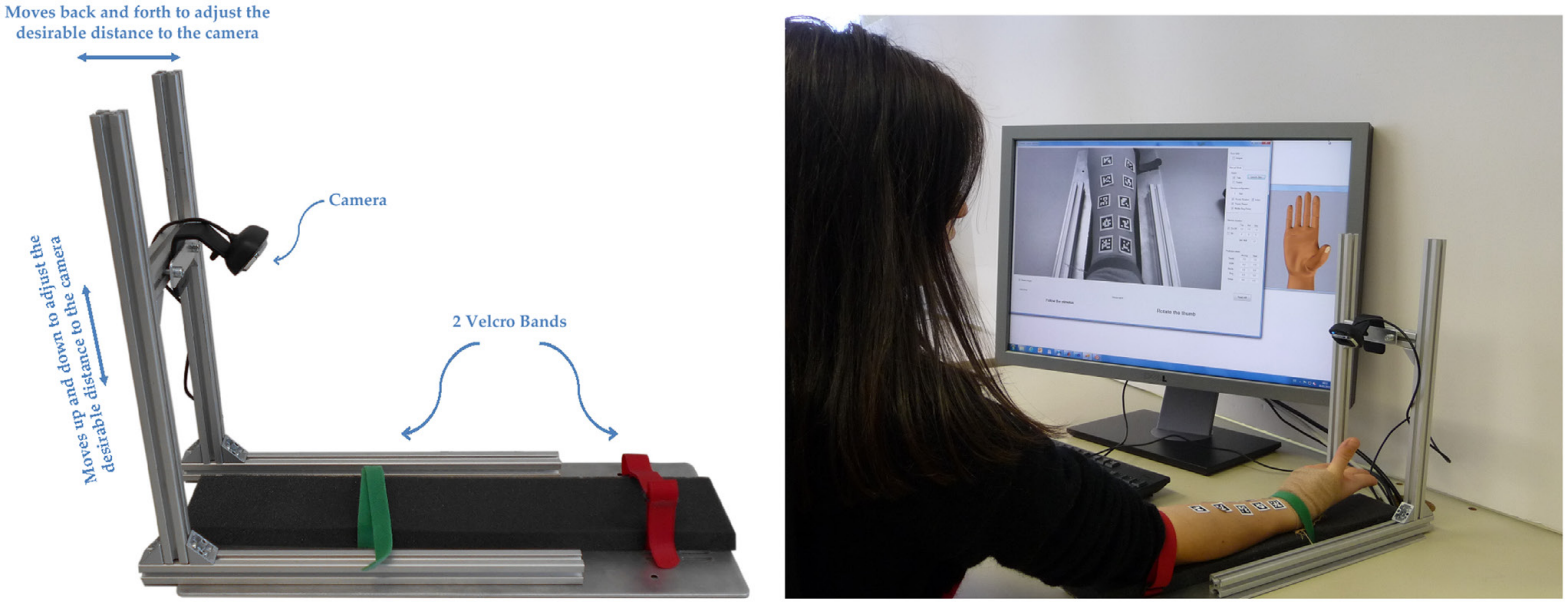
**Left**: the experimental setup, consisting of an off-the-shelf webcam and a rig to which the forearm of a human subject can be fixed. The red arrows depict the camera reference frame, in which the marker transformations are calculated. **Right**: data acquisition while the subject follows the stimulus on the monitor.

In this paper, we present an initial experiment targeting this idea. A standard web camera was placed directly above the forearm of an intact subject, on which 10 AprilTags had been fixed (see Figure 1, right). By tracking the positions of the tags and synchronizing their motion to a visual stimulus that was presented to the subject, we were able to obtain a set of position measurements, some of which would highly correlate to the stimulus itself. We then used two standard machine-learning approaches, namely, a linear one (Ridge Regression, RR (Hoerl and Kennard, 1970)) and a non-linear one (Ridge Regression plus Random Fourier Features, RR-RFF (Rahimi and Recht, 2008b)), to try and map the signals extracted from the tags to the stimulus itself. We also tested the system in conditions of increasing difficulty: using only minimal and maximal activations to predict the intermediate finger position values, artificially changing the contrast and luminosity of the images, blurring them, and limiting the tracking to the markers placed on the proximal part of the forearm. In all cases, the results are highly promising, showing an average error (root square mean error normalized over the signal range) in the reconstruction of the finger positions in the range of 0.05–0.22. Indeed, this approach also compares favorably with previous approaches already used to solve the problem – a detailed numerical comparative analysis can be found in this work.

This work can be considered the extension of a previous preliminary result presented at ICORR 2015 (Nissler et al., 2015), in which a linear machine-learning method is applied. This work builds up on this, incorporating a non-linear method and comparing both methods with state-of-the-art human–machine interfaces as well as testing the robustness of the methods.

## 2 Materials and Methods

### 2.1 Experiment Description

#### 2.1.1 Setup

For the experiments, an off-the-shelf computer (6-GB RAM, Intel Xeon 2.8-GHz CPU) and camera (diagonal angle of view of 68.5°, fixed focus, resolution of 1280 × 720 pixels, and a frame rate of 15 frames/s) are used. In this experiment, we assume a fixed relation of the camera to the forearm, which was achieved by developing a simple setup (Figure 1, left). It can be adjusted to individual subjects and stabilizes the forearm during the experiments with the aid of velcro straps.

The software mainly consists of three components: a *three-dimensional hand model*, which is used to display a visual stimulus on a computer monitor; a *graphical user interface*, which allows the examiner to set the appropriate parameters for the experiments (degrees of freedom, number of repetitions for each task, duration of each repetition, and stimulus signal); and which connects to the hand model by User Datagram Protocol (UDP). Furthermore, it is used to display and save the images from the camera and at the same time record the stimulus values using a unique identifier for each frame and a time stamp. The *tag detection software* is a C++ version of the original AprilTag detection algorithm, as shown in Olson (2011), which offers 6D feature localization (position and orientation) relative to the camera from a single camera image. In Figure 1, right, the stimulus hand model is shown on the right, and the graphical user interface giving feedback is shown on the left of the computer screen.

#### 2.1.2 Participants

The goal of the experiments was to evaluate the performance of the approach achieved by human subjects. Ten healthy human subjects, two females and eight males (27.3 ± 5.66 years old, mean ± SD) (Table 1), two left-handed and eight right-handed, participated. The circumference of the forearm of the subjects was measured and averages 25.7 ± 1.7 cm (mean ± SD) (Table 1). All subjects were informed, both in writing and orally, about the procedure and possible risks. To the best of our knowledge, the only possible risk, because of the nature of our experiments, is an allergic reaction caused from the contact of the skin with the plastic velcro bands or the paper stickers, which were placed on the forearm. The experiments were performed according to the WMA Declaration of Helsinki, were preliminarily approved by the Ethical Committee of our Institution, and all subjects gave written informed consent before each experiment began.

**Table 1:** **Age and arm circumference of subjects**.

	Average	SD	Min	Max
Age (years)	27.3	5.66	21	42
Arm circumference (cm)	25.7	1.7	23.5	28.5

#### 2.1.3 Experimental Protocol

Before starting the experiments, the camera was calibrated using the Matlab Camera Calibration Toolbox (Bouguet, 2004), in order to obtain the intrinsic camera parameters needed for the AprilTag localization. This only needs to be done once because of the fixed focus of the camera. For the purpose of data acquisition, each subject sat on an adjustable office chair, maintaining an upright posture. The AprilTag stickers were placed on the ventral side of the forearm in a quasi-random alignment, 5 rows with 2 stickers per row, trying to cover the camera-visible area of the forearm. The forearm was affixed with straps.

Each subject was then asked to move her or his fingers according to the movements performed by the 3D hand model (Figure 1, right). The 3D hand model movements act as a visual stimulus which the subject has to follow as closely as possible. Each stimulated movement ranged from a relaxed position to a full flexion or rotation through a square–sinusoidal curve. The movements instructed were as follows: a complete flexion of the thumb around the first axis of the carpometacarpal joint (Hollister et al., 1992) and back; a complete flexion of the thumb around the second axis and back; a complete flexion of the index finger and back; and a complete flexion of the little, ring, and middle fingers together and back. These movements are hereafter denoted as, in turn, thumb rotation (thumb abduction), thumb flexion, index flexion, and combo flexion. Each flexion lasted 5 s, with 3 s in between for rest. This sequence (a “session”) was repeated five times. The whole experiment lasted about 10 min, including preparation and briefing of the subject.

### 2.2 Data Processing and Prediction

#### 2.2.1 Data Preprocessing

**Figure 2:**
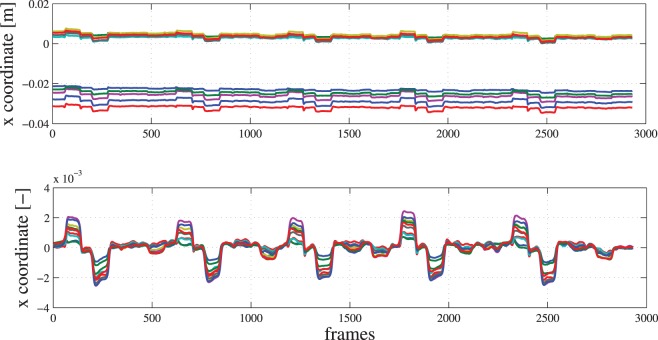
**Raw (upper) and filtered (lower) data of the *x* component (in meters) in the camera reference frame of ten tags for a typical subject**. The *x* axis shows the frame number of the video sequence.

After the frames were captured and saved, the AprilTag locations in every frame were calculated. This is an offline step, relatively time consuming since the frequency of frames processing is around 3.5 fps. Taking into account that a dataset is roughly 3500 frames, tag detection can last up to 20 min. For every dataset, the AprilTag detection algorithm produced a file with a frame ID and the corresponding translation of each marker in the camera reference frame, expressed in meters or radians (*x*, *y*, and *z*: yaw, pitch, and roll). The DC and high-frequency component of each signal were removed, and all signals were centered around zero by applying a second-order Butterworth bandpass filter (lower cutoff frequency of 0.01 Hz and higher cutoff frequency of 0.5 Hz). Typical data used as input to the regression model before and after filtering are shown in Figure 2, each color corresponding to one specific tag and coordinate.

NB: Only typical data for one degree of freedom (translation in *x* direction in the camera reference frame) are shown in Figure 2, but full six-dimensional data are used as input to the machine-learning algorithms. Undetected markers were conservatively replaced by their last known position. The ground truth was the time profile of the visual stimulus that the subject had to follow, ranging from 0 (relaxed position) to 1 (full flexion or rotation). We intentionally avoided the usage of any ground-truth sensors, such as an instrumented glove, in order to keep the setup as light as possible. This technique has already successfully been used in our previous work (Castellini and Sierra González, 2013).

#### 2.2.2 Regression Model

A map from the AprilTags positions and orientations to the visual stimulus (one when activated and zero otherwise) was built using a regression model developed in Matlab. We applied *Ridge Regression* (RR) and *Ridge Regression with Random Fourier Features* (RR-RFF) to build the regression models; grid search for the optimal values of the hyperparameters (*λ* in RR, *λ* and σ in RR-RFF) was performed by computing the normalized root mean squared error (NRMSE). The mathematical details of the two methods are out of scope here; the interested reader should refer to Gijsberts et al. (2014), where both methods were used and demonstrated using surface electromyography. Briefly, RR (Hoerl and Kennard, 1970) builds a linear map from an input space to an output space by evaluating an optimal weight vector *w* minimizing the sum of the Mean Squared Error between the predicted and real data and a regularization term: *w* = arg min*_*w*_* (*y* − *Xw*)^2^ + *λ*||*w*||^2^. The optimal weight vector turns out to be *w* = (*X^T^X* + *λI*)^−1^ *X^T^y*, where *I* is the identical matrix and *λ* > 0 is a regularization coefficient. As opposed to RR, RR-RFF (Rahimi and Recht, 2008a,b) is essentially a Least-Squares Support-Vector Machine (Boser et al., 1992; Rifkin et al., 2003) in which, instead of the classical Gaussian kernel, a finite-dimensional approximation of it, based upon Fourier coefficients, is used; from another point of view, it can be seen as a non-linear, finite-dimensional extension to RR. In general, kernelized RR is a regularized least-squares method, in which the input space is projected onto a higher order *feature space* where linear regression is supposedly possible; in the case of RR-RFF, a direct non-linear mapping *ϕ* can be built, such that each input sample can be simply replaced with its projection onto the feature space, *ϕ*(*x*). The previous equation can then be rewritten as *w* = arg min*_*w*_* (*y* − Φ*w*)^2^ + λ||*w*||^2^, and the optimal weights are evaluated as *w* = (Φ*^*T*^*Φ + *λI*)^−1^Φ*^T^y* where Φ = *ϕ*(*X*). In RR-RFF, we consider a Fourier space with frequencies *ω* ~ *N*(0, *σ*^2^*I*) and phases *β* ~ *U*(−π, π), such that $\phi (x)=\sqrt{\frac{2}{D}}cos(X\Omega +\beta )$, where *D* is the dimensionality of feature space (set at 500 in our experiments).

#### 2.2.3 Validation

Data obtained from each subject were treated independently. Five different ways of testing the approach were considered:

• *The 10-fold cross-validation with shuffling using all values*. We split the whole dataset (using the intermediate values as well) in ten equal folds randomly, and we use nine for training and one for testing. For each degree of freedom, optimal values of the hyperparameters are calculated.
• *The 10-fold cross-validation with shuffling using only on–off values*. We keep only the frames where the stimulus is activated, and we split into ten equal folds randomly. We use nine for training and one for testing. For each degree of freedom, optimal values of the hyperparameters are calculated.
• *On–off values for training and then predict the intermediate values*. We train the system using all the on–off values and we test by predicting all the intermediate values, while applying a grid search for *λ* and *σ* without cross-validation, due to the limited size of our dataset.
• *The 5-fold cross-validation without shuffling using all values*. We split the whole dataset (using the intermediate values as well) in five equal consecutive folds without shuffling, so as to have one repetition in each fold. We then train with 4 repetitions and test with one. For each degree of freedom, optimal values of the hyperparameters are calculated.
• *The 5-fold cross-validation without shuffling training with on–off and predict all values*. We split the whole dataset (using the intermediate values as well) in 5 equal consecutive folds without shuffling. We train with the on–off values of 4 repetitions and test with all values of the 5th repetition. For each degree of freedom, optimal values of the hyperparameters are calculated.

In order to estimate the performance, the results are post-processed by evaluating certain metrics, i.e., the mean values over the subjects and the SDs.

A grid search is then conducted to determine the optimal values for the hyperparameters of each method. In RR, we just need to optimize the regularization term *λ*, and for that purpose, we create a logarithmic space of ten equally spaced exponents which range between [−6, 0]. In RR-RFF, a grid search in order to optimize both SD *σ* and the regularization term *λ* is performed. The space for the grid search is a combination of the ten exponents of the logarithmic space of *λ*, as calculated in Ridge Regression, with ten equally spaced exponents for σ in the interval [–3, 3]. This makes a grid of one hundred different pair combinations. The spaces were chosen after several trials with bigger intervals. The metric used for the optimization is the minimization of the normalized root mean squared error, which in our case is the same as the root mean squared error since the output varies between [0, 1]. The search is performed in combination with the cross-validation methods presented above. The result is one optimal value per degree of freedom for each subject in each method. The time needed to complete the grid search for all degrees of freedom per subject in each method is presented in Table 2. RR, as expected, is much faster than RR-RFF since the evaluation is performed for ten different values instead of one hundred of the latter. Another remark one can make for the time performance of the five different learning methods is that the *train on/off, predict intermediate* is more efficient, which is also anticipated taking into consideration that it is the only method that does not use cross-validation. Thus, the optimal values are calculated after ten (in RR) or one hundred (in RR-RFF) evaluations in contrast to the others, where fifty and five hundred (5-fold cross-validation) or one hundred and one thousand (10-fold cross-validation) evaluations are needed, respectively.

**Table 2:** **Time for grid search in seconds for all training methods with either Ridge Regression (RR) or Ridge Regression with Random Fourier Features (RR-RFF)**.

	RR	RR-RFF
10-fold all values	1.76	712
10-fold on/off values	0.80	396
Train on/off, predict intermediate	0.13	49
5-fold all values	0.76	332
5-fold train on/off, predict all	0.48	183

## 3 Results

### 3.1 Parameter Optimization

As described in the previous section, optimal values for each degree of freedom and each subject individually were determined. In RR, little or no difference in the hyperparameter *λ* can be found when using 10-fold cross-validation either with all or only the on–off values. In these two methods, *λ* can be set at 10^−6^ for all degrees of freedom and all subjects. Unfortunately, this is not the case for the other methods, for which values vary for each degree of freedom. Mean values can deviate up to ±10^2^ (index flexion in *train on–off, predict intermediate*) in those cases. In RR-RFF, all optimal mean values for *λ* fluctuate around 10^−2.5^. However, this does not allow us to set *λ* at a specific value, as the SD is much higher. On the other hand, most optimal values for *σ* are centered around 10^0.5^, with a small SD of about ±10^0.5^.

### 3.2 Accuracy

**Figure 3:**
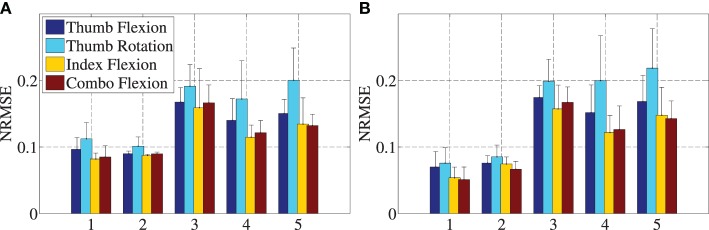
**Mean values of normalized root mean squared errors and SD for Ridge Regression (A) and Ridge Regression with Random Fourier features (B)**. Numbers 1–5 on the *x*-axes are in turn *10-fold cross-validation with shuffling using all values*, *10-fold cross-validation with shuffling using only on–off values*, *On–off values for training and then predict the intermediate values*, *5-fold cross-validation without shuffling using all values*, and *5-fold cross-validation without shuffling training with on–off and predict all values*.

Figures 3A,B depict the errors of each method in both linear and non-linear regression. Starting with Ridge Regression errors (Figure 3A), *10-fold cross-validation* shows the best performance. In the worst-case scenario when *training with on–off and predicting the intermediate values*, the mean errors range from 0.15 to 0.2, showing the worst performance. Side by side, *5-fold cross-validation* seems to have lower accuracy than *10-fold cross-validation*, with mean errors varying from 0.11 to 0.2. In methods where the system is asked to predict the output of a “black box,” such as the repetition-wise cross-validations or the *training with on–off and predicting the intermediate values*, the errors get almost doubled. Overall, the non-linear method does not particularly improve over the linear one. Student’s two-tailed *t*-test was applied to analyze the significance of the results; the corresponding *p*-values are always larger than 0.05, showing that no statistically significant difference exists between the performance of Ridge Regression and Ridge Regression with Random Fourier Features. Furthermore, the adequate accuracy of the linear method favors the assumption that there is a quasi-linear relationship of the tags movement to the finger movements.

### 3.3 Comparison with Previous Methods

The accuracy obtained by OMG can be directly compared with previous results obtained in a recent paper (Ravindra and Castellini, 2014), in which three further human–machine interfaces, namely, electromyography (EMG), ultrasound (US), and force myography (FSR), were compared in an experiment very similar to the one described here. Single-finger flexions following a visual stimulus were stimulated from ten intact subjects, while gathering fingertip forces. Five repetitions at 80% of the maximal force and five repetitions at 15% of the maximal force were performed. The method followed for data handling and splitting is the same as our *5-fold training with on/off values, predict all* method, while for the target output, either the data from a ground-truth force sensor or the visual stimulus (as is the case here) were used. Performance was evaluated using the Normalized Root Mean Square Error as well. In order to compare the accuracy of OMG to those obtained in that paper, we compare the *5-fold training with on/off values, predicting all* in both linear and non-linear regression to the results obtained in the published work from the five repetitions at 80% of the maximal force when stimulus values are used. Additionally, the errors from little, ring, and middle fingers are averaged to imitate the combination flexion followed in our work. For the sake of completeness, the errors of both FSR iterations, attached with the EMG as well as with the US are presented (called FSR 1 and FSR 2), although no statistically significant difference was observed (a Student two-tailed *t*-test obtains *p*-values always larger than 0.05 except for one single case).

**Figure 4:**
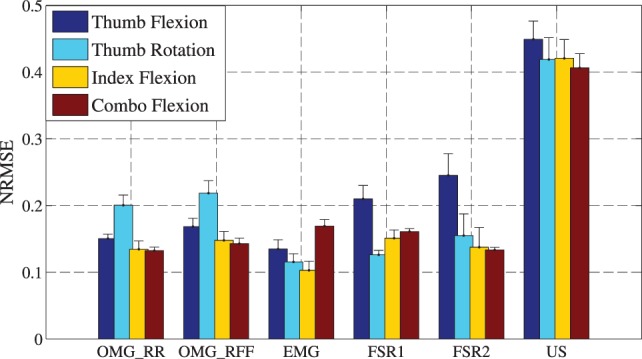
**Comparison of OMG with Ridge Regression (*OMG_RR*) and OMG with Ridge Regression and Random Fourier Features (*OMG_RFF*) with previous methods, electromyography (*EMG*), force myography (*FSR*), and ultrasound (*US*), showing the mean errors and the SEM of every method**.

Figure 4 illustrates the NRMSE for every degree of freedom averaged over ten subjects. Please note that the error bars in Figure 4 show the SEM instead of the SD used before. This was done to be congruent with the results presented in Ravindra and Castellini (2014).

#### 3.3.1 Significance Analysis

A standard Student’s two-tailed *t*-test was evaluated comparing our methods to previously examined methods. The significance analysis for the Ridge Regression method (*OMG with RR*) shows a very significant difference compared to Ultrasound methods (*p*-value almost 0 for all degrees of freedom). For OMG thumb flexion, a strong significant difference is found as well compared to FSR methods. A less strong, but still significant, difference is observed when index flexion with OMG is compared to EMG methods and when combo flexion with OMG is compared to both EMG and FSR methods. All other methods show no significant difference (*p*-value is always >0.05).

Analyzing OMG with RR-RFF, again, the difference to the US method is very strong for all degrees of freedom. The index flexion in OMG compared to EMG method shows as well a very high difference, OMG thumb flexion compared to EMG and FSR 2 methods a still significant difference. In all other cases, there is no significant difference.

### 3.4 Robustness Evaluation

#### 3.4.1 Robustness to Optical Disturbances

**Figure 5:**
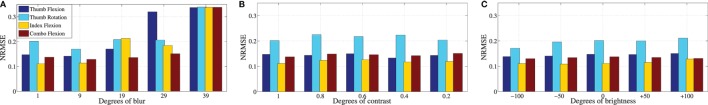
**Error (NRMSE) for one example subject using Ridge Regression with 5-fold cross-validation, training with on/off values and testing with all values, for (A) increasing blur, where the number on the *x*-axis corresponds to the kernel size of the Gaussian blurring; (B) decreasing contrast (*α*), where 1 is full contrast and 0.2 corresponds to 20% of the original contrast value; and (C) changing brightness (*β*), where 0 corresponds to the original brightness value, negative values correspond to underexposure of the image and positive values to overexposure**.

In order to evaluate the robustness of the marker detection to changing light conditions, the images of one example subject were artificially blurred, the contrast was decreased, and the brightness was changed (to overexposed and underexposed images). Figure 5 shows the resulting errors for the Ridge Regression case, namely, in Figure 5A, the effect of a Gaussian blurring with a kernel of size up to 29 pixels, in Figure 5B, the effect of a contrast change, and in Figure 5C, the effects of a change in overall brightness. The change in contrast *α* and brightness *β* is defined by

(1) $$\tilde{f}(x,y)=\alpha \times f(x,y)+\beta$$

where $\tilde{f}(x,y)$ stands for the changed brightness or contrast value of the original value and *f*(*x*, *y*) for the pixel at position (*x*, *y*).

As one can see, an increasing blur negatively affects the quality of the tag detection, thereby increasing the overall error of the regression. On the other hand, the accuracy is not significantly affected by changing brightness or contrast; in some cases, a lower brightness even decreases the error, which means that the original captured images were probably slightly overexposed.

#### 3.4.2 Robustness to Reduced Number of Markers

**Figure 6:**
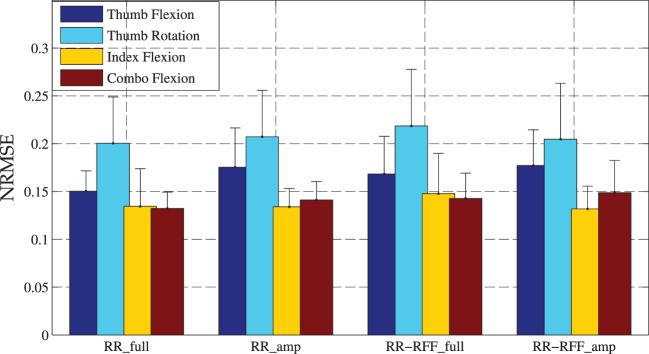
**Comparison of OMG with Ridge Regression and OMG with Ridge Regression and Random Fourier Features for intact subjects (RR_full, RR-RFF_full) to *simulated* amputees by trimming the dataset (RR_amp, RR-RFF_amp)**.

We further analyzed the performance of the system in a closer-to-application scenario, namely, by considering as input data only the six most proximal markers. Here as well, we use 5-fold cross-validation without shuffling and training with only on–off values, which is the most realistic validation method with respect to the case of amputees. Figure 6 shows that the error does not significantly increase, even in this setting.

## 4 Discussion

In this paper, we have the feasibility in principle of a novel human–machine interface for the disabled, aimed at non-invasively and cheaply detecting the intent of an upper limb injured patient. We call the technique optical myography (OMG). We have focused on the case of hand amputees, showing that finger movements can effectively be reconstructed by looking at the human forearm. As ground truth, we used goal-directed stimuli, potentially enforcing the feeling of agency (Limerick et al., 2014) and embodiment (Marasco et al., 2011) enjoyed by the subject, making the experience smoother, easier, and more exciting, and probably leading to better results especially as the training goes on along time. It is a well-known fact that human subjects can adapt to an environment or task that is novel from the sensorimotor point of view (Botvinick and Cohen, 1998; Marini et al., 2014), and this is an effect which should definitely be exploited when it comes to this kind of interfaces.

As far as the accuracy of OMG is concerned, the *10-fold cross-validation* method shows the best performance as expected, since random shuffling of data ensures that each machine-learning method is trained on a uniformly sampled probability distribution; in practical terms, in this scenario, the system always has information about the behavior of the tag movements throughout the whole experiment, and hyperparameters can be optimized more efficiently using data samples from all sessions. In the worst-case scenario when *training with on–off and predicting the intermediate values*, the mean errors range from 0.15 to 0.2, showing the worst performance. The *5-fold cross-validation* seems to have lower accuracy than *10-fold cross-validation* with mean errors varying from 0.11 to 0.2 – this is probably due to slight movements of the forearms and to the different behavior among repetitions. Thumb rotation seems to have the worst performance, which can be explained by the fact that the muscles responsible for this movement are either deep in the forearm or intrinsic in the hand. On the other hand, index and combo flexion, activated by the *M. Flexor Digitorum Superficialis* and *Profundus*, can be easily detected. The *10-fold cross-validation* method shows the best performance as expected, since random shuffling of data ensures that each machine-learning method is trained on a uniformly sampled probability distribution; in practical terms, in this scenario, the system always has information about the ­behavior of the tag movements throughout the whole experiment, and hyperparameters can be optimized more efficiently using data samples from all sessions. In the worst-case scenario when *training with on–off and predicting the intermediate values*, the mean errors range from 0.15 to 0.2, showing the worst performance. The *5-fold cross-validation* seems to have lower accuracy than *10-fold cross-validation*, with mean errors varying from 0.11 to 0.2 – this is probably due to slight movements of the forearms or to the different behavior among repetitions. Thumb rotation seems to have the worst performance, which can be explained by the fact that the muscles responsible for this movement are either deep in the forearm or intrinsic in the hand. On the other hand, index and combo flexion, activated by the *M. Flexor Digitorum Superficialis* and *Profundus*, can be easily detected. Overall, even in the worst-case scenario (recall Figure 3), OMG is able to predict the finger positions with an average error in the range of 0.05–0.25. Comparison with accuracy results obtained in a similar experiment (Ravindra and Castellini, 2014) reveals that the performance obtained by our visual tracking system is in the same range of sEMG and Force-Sensing Resistors, that is, the two interfaces tested in that work. Other similar works, in which EMG is used for regression on finger position (for example, Ameri et al. (2014)), show similar values of accuracy. We further tested our method for robustness against optical disturbances (changes in focus, luminosity, and contrast), showing that the system is robust to such problems. A final test for robustness was conducted by considering only the AprilTags fixed on the proximal section of the forearm, a situation which “simulates an amputee,” since such patients would be able to only wear some of the markers depending on the length of the stump. In this case, too, our system provides a reasonable accuracy.

With respect to competitor techniques (pressure, EMG, and so on), OMG has the advantage of needing almost no subject preparation, i.e., no sensors need to be placed and kept fixed on the subject’s forearm – it is only necessary to place markers on it. Moreover, the hardware used to gather the images is very cheap, consisting of only a consumer-grade webcam and an off-the-shelf computer running the software obtaining the camera in out and parallel giving feedback to the user. The fact that we used the AprilTags ensures a certain stability with respect to illumination changes and motion blur. Notice that here too, as is customary in similar literature, we explicitly *did not target any anatomical features of the forearm* but rather placed the AprilTags uniformly on the forearm skin. This is motivated by the fact that it considerably simplifies the preparation of the subject, and at the same time makes the approach more realistic, since the anatomy of stumps varies across subjects due to the kind of amputation.

Among the main disadvantages of OMG in the present setting: first and foremost, the fact that (in our experiment) the subject’s forearm was fixed in a determined position, in order to avoid forearm movement which was not correlated to the muscle bulgings. Second, the algorithm detecting the positions of the AprilTags cannot yet run online, because the camera frequency is higher than the frequency of the detection. This could be solved in future trials by parallelization. Third, in the current setup, the tags were placed only on the ventral side of the forearm due to the simplicity of the setup; this is probably the reason why the hardest degree of motion was the thumb rotation, whereby the corresponding muscles are at the posterior side, like mentioned before (Section 3.2). In general, occlusion of the tags, as in every marker-based visual tracking problem, is going to be one of the main hurdles and will need to be solved. It would be thus favorable to have a markerless system. However, as mentioned before, the human skin offers only very little texture making feature detectors for natural landmarks very problematic. Another approach would be a depth sensor, such as the Microsoft Kinect, which is right now prevented by the low precision these sensors offer.

Interestingly, it seems (recall Figure 3) that RR performs comparably, if not even a little better, to RR-RFF, which builds a non-linear model, indicating that, to some extent and for the experiment considered, there is a linear relationship between the positions of the AprilTags on the forearm and the position of the fingers. This could be related, although we cannot claim any result about this, to the linearity of the relationship between finger positions and *ultrasound images* shown in Castellini and Passig (2011). Although ultrasound images are of a completely different nature, they still visualize (inner) structures whose positions are directly related to muscle deformations, which in turn related to the intended level of activation. Clearly, that the relationship we have tried to model here is linear comes as a big advantage, given that RR is one of the simplest and fastest forms of machine learning available. Notice, however, that linearity probably holds only as long as, as it is the case here, the forearm stands in a fixed position with respect to the camera. This is the subject of future research.

As we envisioned in the Introduction, we plan to extend this work to realize a motion tracking system for an amputee’s stump, able to reconstruct the missing limb in a virtual world. Clearly, the challenge is formidable. First, one must track in real time the stump itself, at least finding its position and orientation; second, a set of markers or natural landmarks must be identified and tracked *on the stump*, and their relative positions compared to detect the deformations of the stump. This also depends on the degree of residual activity still found in the subject’s residual muscles. The results presented in this paper at least show that in principle this might suffice to perform the desired reconstruction.

Several assumptions made in this paper have to be addressed in order to make the usage in a Virtual-Reality scenario feasible. In our proof of concept, the forearm of the subjects is fixed. In order to provide a realistic AR/VR impression to the user, the prototypical setup shown in this paper has to be extended to a mobile system, which can be worn by an amputee. The hardware, computational power, and high-resolution camera needed are nowadays available in wearable devices. Even superior would be not attaching the camera to the arm, but looking at the arm “freely,” with a static camera and the arm moving in front of it. The benefits of such an approach are obvious, but the challenges are huge: for example, such a system would need a robust motion detection and tracking for the arm itself, because the assumption we take in our prototype, that the camera is fixed relative to the forearm, no longer applies. However, such a system would not only allow a hand amputee to interact naturally in a VR but also VR therapy would be possible, such as the treatment of phantom pain.

Another interesting experiment would be to compare our approach with hand motion ground truth obtained by a hand tracking system like shown in Sharp et al. (2015) or the commercially available Leap Motion system (www.leapmotion.com), which offers a relatively high precision and robustness (Weichert et al., 2013).

## Author Contributions

CN: acquisition and analysis of captured data, implementation of algorithms, and final approval of the work. NM: contributed to the acquisition and analysis of captured data, the implementation of machine-learning algorithms, and final approval of the work. CC: contributed to the interpretation of the recorded data, revising the work, and final approval of the work.

## Conflict of Interest Statement

The authors declare that the research was conducted in the absence of any commercial or financial relationships that could be construed as a potential conflict of interest.
